# Understanding the link between obesity and headache- with focus on migraine and idiopathic intracranial hypertension

**DOI:** 10.1186/s10194-021-01337-0

**Published:** 2021-10-10

**Authors:** Connar Stanley James Westgate, Ida Marchen Egerod Israelsen, Rigmor Højland Jensen, Sajedeh Eftekhari

**Affiliations:** Danish Headache Center, Department of Neurology, Rigshospitalet- Glostrup, Glostrup Research Institute, University of Copenhagen, Nordstjernevej 42, 2600 Glostrup, Denmark

## Abstract

**Background:**

Obesity confers adverse effects to every system in the body including the central nervous system. Obesity is associated with both migraine and idiopathic intracranial hypertension (IIH). The mechanisms underlying the association between obesity and these headache diseases remain unclear.

**Methods:**

We conducted a narrative review of the evidence in both humans and rodents, for the putative mechanisms underlying the link between obesity, migraine and IIH.

**Results:**

Truncal adiposity, a key feature of obesity, is associated with increased migraine morbidity and disability through increased headache severity, frequency and more severe cutaneous allodynia. Obesity may also increase intracranial pressure and could contribute to headache morbidity in migraine and be causative in IIH headache. Weight loss can improve both migraine and IIH headache. Preclinical research highlights that obesity increases the sensitivity of the trigeminovascular system to noxious stimuli including inflammatory stimuli, but the underlying molecular mechanisms remain unelucidated.

**Conclusions:**

This review highlights that at the epidemiological and clinical level, obesity increases morbidity in migraine and IIH headache, where weight loss can improve headache morbidity. However, further research is required to understand the molecular underpinnings of obesity related headache in order to generate novel treatments.

## Introduction

Obesity is a global health issue, affecting over half a billion individuals and is defined by the World Health Organisation as having a body mass index (BMI) > 30 kg/m^2^. Obesity constitutes a large disease burden in  terms of socioeconomic costs and patient morbidity[1]. This is a consequence of obesity being a precipitating factor in a myriad comorbidities including type 2 diabetes mellitus, cancer, hypertension and premature death[2, 3]. Underlying these comorbidities is biochemical and hormonal dysfunction driven by excess adiposity and low-level chronic inflammation. Given this systemic biochemical phenotype, obesity affects every aspect of the body including the nervous system .

Headache disorders such as migraine and the metabolic disease Idiopathic intracranial hypertension (IIH), have been associated with obesity. Migraine affects over a billion individuals, where migraine is associated with a high level of disability[4]. Epidemiologically, it is clear that migraine is over-represented in the obese population. However, the causes underlying this association have been considered controversial and under investigated. The vast majority of IIH patients are phenotypically obese females of reproductive age, where headache is the primary co-morbidity, thus IIH forms a case study of a disease of pure obesity and headache. However, although obesity and headache are a clear commonality, whether these are linked pathophysiologically is subject of debate. Although there is a link between obesity and these headache disorders, the underlying mechanisms driving the headache remain elusive.

In this narrative review, we have reviewed the clinical and epidemiological evidence for a link between obesity and the headache in migraine and IIH. We have explored the phenotypes, both clinical and molecular, to identify the links between obesity, migraine and IIH. Furthermore, the current review evaluates the pre-clinical evidence for alterations in headache like behaviour and the potential molecular underpinnings of these behavioural changes in rodents. Finally, the potential role of obesity on raised intracranial pressure (ICP) in the development of headache in migraine and IIH is discussed.

## Methods

A narrative review was conducted where both clinical and preclinical evidence for a mechanistic link between obesity and the headache in migraine and IIH was evaluated. English language papers in both PubMed and Google Scholar were consulted for this review between its inception and September 2021, where both primary research and review articles were considered. Keywords utilised in this review included but were not limited to: ICP, headache, obesity, CGRP, migraine and IIH.

### Obesity: Epidemiology, pathogenesis and treatments

Obesity is a disease on pandemic proportions, affecting over 650 million individuals worldwide, where the prevalence is highest in the developed world and is associated with low socio-economic status[5]. Obesity is associated with excess adiposity and increases the risk of developing serious comorbidities including type 2 diabetes mellitus, cancer, musculoskeletal diseases, cardiovascular diseases, metabolic syndrome, mental health issues and premature death[2, 3, 6]. However, obesity is a modifiable disease where BMI reduction confers improvement of the comorbid conditions and can be curative[6].

Obesity is a complex multifactorial disease where several components contribute to its pathogenesis. It is clear that obesity has a strong genetic component, where a genome wide association study identified that polymorphisms in multiple nervous system genes are associated with the development of raised BMI in European men and women[7]. Moreover, it has been demonstrated that polymorphisms associated with a lean phenotype also exist[8]. As such, obesity susceptibility can be described as a polygenic risk: where an accumulation of polymorphisms increases the risk of developing obesity. Additionally, environment plays a strong role in the development of obesity, where lifestyle, availability of calories and socio-economic status independently increase the risk of developing obesity in western society[9]. All of these components increase the risk of excess lipid accumulation in adipocytes, where excess adipocyte lipids cause a pro-inflammatory state that promotes the systemic manifestation of obesity[10].

There are various therapies for obesity, all of which focus on weight loss, which is the only disease modifying treatment for obesity. However, these therapies are often hampered by low efficacy and side effects where long-term remission can be difficult to maintain. Furthermore, these therapies are also multifunctional as they often treat co-morbidities of obesity[11]. Pharmacotherapy for obesity is becoming increasingly common, novel drugs are entering the market and is reviewed by Velazquez et al. here[11]. In brief, obesity pharmacotherapeutics aim to either directly modulate the processing of macronutrients or aim at altering gut-neuropeptides thus modulating systemic metabolism and feeding behaviour[11]. Weight loss can also be achieved surgically, where various forms of bariatric surgery are utilised[12]. Although these provide a mechanical alteration as to how food is processed post-prandially, the primary mode of weight loss is mediated through changes in gut-neuropeptides[12]. This rebalancing of gut-neuropeptides alters feeding behaviour and the metabolic metabolism, thus facilitating weight loss[12].

Obesity is associated with insulin resistance, dyslipidaemia, an altered sexually dimorphic endocrine phenotype and increased circulating pro-inflammatory cytokines including IL-1β, leptin and IL-6 and decreased circulating levels of anti-inflammatory cytokines such as adiponectin[13–15]. Consequently, obesity can be defined as both a metabolic disease and a state of chronic low-grade inflammation. Obesity is a disease with multimodal effects on physiology: adversely affecting the vasculature, where migraine pain may originate from the cerebral vasculature[16]. Furthermore, the chronic state of inflammation in obesity could be linked to the proposed role of neuroinflammation in migraine pathogenesis[16]. Given these, it is perhaps unsurprising that there is a link between migraine and obesity.

### Migraine: Epidemiology, features and treatments

Migraine is a common disease, estimated to affect 1.04 billion individuals, where 18.9 % of females and 9.8 % of males have a migraine diagnosis[4]. Similarly to obesity, migraine is associated with a lower socio-economic background[17]. Clinically, migraine is well defined by the ICHD-3 criteria[18]. Cutaneous allodynia, the perception of a painful stimulus that otherwise would not be painful, is present in 63.2 % of patients with migraine. The severity of cutaneous allodynia is positively associated with the severity and frequency of migraine attacks and is a likely predictor of migraine chronification[19, 20]. However, migraine is not only a headache disorder, migraine patients experience premonitory and post monitory symptoms including tiredness and difficulty concentrating around the migraine attack, further contributing to disease morbidity[21]. Given the symptomology and commonality of migraine it comprises one of the largest disability burdens of any disease, second only to lower back pain[22].

Various therapeutics exist for the treatment of migraine, which either aim at treating migraine pain (abortive therapy) or preventing the development of a migraine attacks (preventative therapy). The primary abortive therapies for migraine are nonsteroidal anti-inflammatory drugs (NSAIDS), these are efficacious for some but are non-specific for migraine[23]. Triptans, 5-HT_1B/D_ receptor agonists, are migraine specific abortive therapeutics[23]. Multiple non-specific drugs are used to prevent migraine and fall into several classes including: antihypertensives, antidepressants, anticonvulsants and calcium channel blockers[23]. More recently, therapeutics that target the underlying migraine pain pathway have come to the market, monoclonal antibodies targeting calcitonin gene related peptide (CGRP) or its receptor with remarkable efficacy[23]. Treatments for migraine, whether abortive or preventative, are only symptomatic treatments as they do no treat the underlying pathology.

### Idiopathic intracranial hypertension: Epidemiology, features and treatments

IIH is metabolic disease defined by raised intracranial pressure (ICP) and papilledema of unknown aetiology[24–28]. IIH is a rare disease, affecting 4.69 per 100,000 in the general population, where the incidence is increasing in line with the increasing incidence of obesity[29]. Indeed, the incidence of IIH is 20 per 100,000 in the obese population, with up to 64 per 100,000 in females[30]. There a several phenotypic presentations of IIH, however the most common is presenting in obese women of reproductive age. Thus, the disease associated with the female gender and obesity is the IIH that will be discussed in this review.

IIH patients present with a plethora of features outside of the diagnostic criteria that are seen in excess to that conferred by obesity. Cognitively, IIH patients have deficits in executive function and attention[31]. Perhaps linked to this, roughly half of IIH patients have obstructive sleep apnoea (OSA), where reduction in OSA following weight loss is associated with improved executive function and attention in IIH[32]. Headache is a predominant symptom in IIH, where over 90 % of patients experience headache. Headache represents the biggest cause of morbidity and disability after the visual components of the disease are resolved[33]. The headache phenotype in IIH has previously been reviewed in detail by Mollan et al.; where the headache phenotype in IIH is predominantly migrainous, thus leading to a state mimicking chronic migraine[34]. Given this evidence, we will consider migraine headache and IIH headache mechanistically synonymous in this review. Likely linked to the chronic headache in IIH, depression is a feature of IIH, perhaps unsurprising given the presence of chronic pain[35]. Indeed, IIH patients report that headache is the primary determinate in their quality of life and once resolved quality of life is much improved[33]. However, the other features of IIH are also reported to reduce their quality of life[36]. The mechanisms underlying the headache in IIH remain unelucidated and has been highlighted as a research focus[37].

Multiple treatments for IIH exist and depend on the severity of papilledema. In cases of fulminant IIH (vision at risk) neurosurgical CSF diversion is used to preserve vision[38]. Otherwise, IIH is medically managed primarily with acetazolamide and secondarily with topiramate[38]. These drugs are proposed to reduce ICP. However, a Cochrane review suggests that there is currently insufficient evidence to prescribe any drug for therapy in IIH[39]. Therapeutically, it has been demonstrated that weight loss improves the headache phenotype in IIH, in tandem with ICP reduction (see Table 1 for representative studies) [40]. However, IIH headache often persists after resolution of raised ICP and papilledema, this would suggest central sensitisation to pain stimuli[34, 41].

**Table 1:** Weight loss treatments and effects on headache in migraine and IIH. Data presented depicts means before and after intervention. BMI = Body mass index, VAS = Visual analogue scale, VRS = Verbal rating scale, MIDAS = Migraine disability assessment

Migraine					
Migraine phenotype	Treatment	Weight change (BMI)	Headache frequency	Headache severity/ medication intake	Ref
Episodic migraine w/out aura	Ketogenic diet 3 months on diet	27.5 to 23.2	(n/month):5.1 to 3.2 (P < 0.05)	Tablet intake (n/month): 4.9 to 2.8 (P < 0.05)	[52]
Episodic migraine w/out aura	Nutritionist assisted diet, 3 months	27.8 to 24.6	(n/month): 6.3 to 4.2 (P < 0.05)	Tablet intake (n/month): 5.9 to 3.8 (P < 0.05)	
Migraine ICHD2	Vegan diet 16 weeks	26.9 to 25.5	(n/week):2.1 to 1.7	VAS: 6.0 to 3.6 (P < 0.0001)	[53]
Migraine ICHD2	Placebo dietary supplements 16 week	26.2 to 26.2	(n/week):2.1 to 1.8	VAS: 4.7 to 4.1	
Migraine w/out aura ICHD2	Gastric banding, 6 month follow-up	42.4 to 34.6	(n/month): 6.0 to 1.0 (P < 0.001)	MIDAS: 21 to 4 (P < 0.001)	[54]
**IIH**					
Phase of disease at study entry	Treatment	Weight change (BMI)	Headache frequency	Headache severity	Ref
> 3 months after diagnosis	Low calorie diet trial 3 months	38.6 to 32.6	(Days/week): 4.4 to 2.1 (P = 0.011)	VAS: 4.2 to 1.9 (P = 0.015)	[55]
Mean 1 year after diagnosis at baseline	Bariatric surgery 1 year after randomisation	44.2 to 35.1	(Days/week): 5.5 to 1.8 (P < 0.001)	VRS: 5.0 to 3.2 (P = 0.002)	[40]
Mean 1 year after diagnosis at baseline	Community weight management 1 year after randomisation	43.7 to 43.1	(Days/week): 5.6 to 4.1 (P = 0.007)	VRS: 5.0 to 4.0 (P = 0.1)	
Newly diagnosed	Routine clinical care 1Y follow-up study	35.5 to 31.8	Daily 86–43 %	VAS: 7.5 to 4.5	[41]

With regards to headache specific treatment, a recent open label study has demonstrated that the CGRP receptor monoclonal antibody erenumab reduces headache disability and morbidity in IIH patients who were already in ocular remission, i.e. with persistent headache[42]. Furthermore, erenumab has been demonstrated to treat headache in active IIH, while raised ICP and papilledema remain[43]. However, given the open label, non-controlled nature of these studies, further investigation is required. These studies suggest that other migraine specific therapeutics should be investigated for efficacy with IIH headache. Additionally, there is consensus that more research is required in understanding and treating headache in IIH[37].

### Epidemiology of migraine with obesity

Given that obesity and migraine are ubiquitous in the population, it is inevitable that individuals will have concurrent obesity and migraine. Indeed the bulk of the literature suggests that there is an association between obesity and increased incidence of migraine[44]. A recent meta-analysis demonstrated that when covariates are taken into account, obesity confers a 21 % increase in migraine diagnosis when compared to non-obese controls[45]. However, this increased risk is low compared to the cardinal comorbidities associated with obesity including type 2 diabetes mellitus (574 %) and hypertension (84 %)[3].

When assessing by age group, it is clear that obesity increases the risk of migraine for patients under 55 years old, particularly for women[46]. This increased incidence in early life is in keeping with the general migraine population and suggests an endocrine component to obesity associated migraine. Obesity effects migraine phenotype, whereby patients with concurrent migraine and obesity are more likely to have a chronic migraine (CM) diagnosis[47]. This suggests that obesity is a risk factor for migraine chronification [47]. Additionally, obesity is associated with a BMI dependent increase in migraine disability, suggesting that obesity increases severity of migraine attacks[48]. Linked to this, obesity has also been demonstrated to be associated with a higher attack frequency, where an apparent dose dependent relationship exists between BMI and headache days[49]. In the paediatric population higher BMI is also associated with increased severity, frequency and disability of migraine, however additional studies are required to confirm this single study[50].

Intuitively, if obesity contributes to the severity of migraine, then weight loss would confer an improvement of symptoms. Indeed, a recent meta-analysis highlights that weight loss, independent of intervention, reduces headache duration, frequency, severity and disability associated with migraine (see Table 1 for representative studies)[51]. This suggests that weight loss is a potential therapeutic strategy for migraine, given that the outcome was independent of starting BMI. Weight loss has also been demonstrated to reduce headache days in paediatric migraine[50]. However, given the low numbers in the weight loss studies, more extensive clinical studies are required in assess the efficacy of weight loss as a migraine treatment. Although weight loss has therapeutic benefits in migraine, weight loss is difficult to achieve and maintain, thus understanding the molecular underpinnings of obesity related migraine is vital in the development of novel therapeutics.

Together, the evidence highlights that obesity confers an increased risk of developing migraine, in particular increasing migraine morbidity. This increased morbidity could partially explain why obese patients are more likely to report migraine: more severe symptoms would necessitate clinical intervention rather than self-management.

### Migraine pathophysiology and obesity

The cause of primary migraine is still unknown, however it is considered to involve genetic factors, activation of the trigeminovascular system, changes in thalamic function and/or dysfunction of the brainstem and release of neuropeptides such as calcitonin gene-related peptide (CGRP)[16]. However, it is clear that in the majority of cases of migraine, it is a combination of these factors that leads to the onset of a migraine attack. Genetics, environment and biochemical factors play a cumulative role altering the threshold for a migraine attack.

Genetics play a large role in the susceptibility of developing migraine, perhaps up to 50 % of the risk[23]. The vast majority of migraine it thought to be mediated by an accumulation of migraine promoting polymorphisms,  i.e migraine susceptibility is a consequence of polygenic risk[56, 57]. However, the polygenic risk is not determinative for migraine. Rather, it lowers an individual’s threshold for migraine, meaning other factors that can precipitate a migraine have the opportunity to prevoke a migraine attack.

CGRP is a potent vasodilatory neuropeptide which also transmits nociceptive information and consists of two variants; αCGRP and βCGRP. αCGRP is predominantly expressed in the central nervous system and βCGRP is primarily expressed in the enteric sensory system. CGRP has been demonstrated to have a significant role in migraine pathophysiology as clinical studies have shown increased levels of CGRP in serum, cerebrospinal fluid, and saliva of migraine patients[58–61]. In support of this, systemic infusion of CGRP can trigger a migraine headache in patients[62]. Therefore, targeting CGRP signalling with small molecule receptor antagonists or with monoclonal antibodies targeted to either the ligand or receptor, have been developed with clinical efficacy[63]. The CGRP receptor consists of the calcitonin receptor-like receptor (CLR) and receptor activity-modifying protein 1 (RAMP1)[64–66]. CGRP can also act and function via the amylin 1 receptor consisting of calcitonin receptor (CTR) and RAMP1, suggesting that CGRP can act at more than one receptor[67, 68]. CGRP and its receptors are widely distributed in the CNS, particularly at regions thought to be involved in migraine pathophysiology, including the trigeminal ganglion (TG), dura mater, brainstem and the cerebellum[69–74].

In the context of obesity, it has been shown that women with obesity had elevated plasma levels of CGRP compared to controls[75]. Preclinical studies have also demonstrated a link between obesity and CGRP. Zucker rats, a model of genetic obesity through hyperphagia related to a non-functioning leptin receptor, have elevated plasma levels of CGRP while pre-obese, although this was not assessed in obese rats[76]. In a specific αCGRP knockout (αCGRP^−/−^) mouse model, it was demonstrated that these mice were protected from diet-induced obesity and maintained normal glycaemic control[77]. This suggests that CGRP has role in metabolic regulation, likely linked to CGRP receptor in the gut[77]. The effect of obesity on the trigeminovascular system has been investigated in rodents, where the basal release of CGRP from meningeal afferents was increased in diet-induced obese rats[78].

The pancreatic hormone amylin is a pro-satiety hormone and is released in the post-prandial state[79]. Amylin shares 25–50 % sequence homology with CGRP and is a well characterised agonist of the CGRP receptor. As with CGRP, levels of amylin are also elevated in obese individuals[79]. Amylin and its analogues are known to induce migraine attacks in patients with migraine[80]. As such, the dual increase of serum amylin and CGRP in obesity could contribute to the increased risk of migraine associated with obesity. Clinically, amylin analogues have been developed to treat type 1 and type 2 diabetes mellitus as well as obesity[81]. Given the capacity of amylin and its analogues to promote headache, the migraine history of a patient should be considered prior initiating amylin receptor agonist therapy to treat diabetes.

Another mechanism that may be involved in migraine is cortical spreading depression (CSD)[82, 83]. CSD consists of a spreading wave of depolarization associated with a reduction of cortical activity and has been related to migraine with aura. CSD in rodents can be evoked by various experimental triggers, and it is used as a model for migraine aura and for evaluating anti-migraine drugs[82, 84–86]. Obese Zucker rats have been demonstrated to have an increased number of CSDs, suggesting obesity affects cortical excitability [87]. Interestingly, in lean rats a CNS infusion of leptin increases the number of CSD’s, linking the higher serum leptin levels in migraine with aura with the proposed increase of migraine with aura in obese females[87–89]. However other physiological changes in obesity may affect the qualities of CSD’s, this requires further investigation.

These studies demonstrate that factors associated with migraine development, are increased in the context of obesity. Consequently, this provides biochemical evidence that obesity could contribute to an increased risk of developing and aggravating migraine.

### Endocrinology and body composition in migraine and IIH

Although it is apparent that obesity is associated with migraine and IIH, the mechanisms underlying this remain unclear. Obesity has profound effects on human metabolism and endocrinology, where the distribution of adiposity dictates the level of metabolic and hormonal derangement[90, 91]. More specifically, abdominal and visceral obesity is associated with more severe metabolic outcomes[90, 91].

Given this, it is curious that abdominal obesity was associated with a higher prevalence of migraine in both men and women below 50 years of age, although women are particularly affected (OR = 1.26)[46]. In addition, at a given BMI, migraine patients with cutaneous allodynia have greater visceral adiposity compared to migraine without cutaneous allodynia. This visceral adiposity present with cutaneous allodynia is associated with increased disability[92]. Assessing the capacity of weight loss to modify cutaneous allodynia in migraine patients would be important to allow additional strategies to manage this disabling feature of migraine. No differences in the pro-inflammatory cytokines TNF-α and IL-6 were observed between cutaneous allodynia and non-cutaneous allodynia patients, suggesting inflammation is not a factor underlying the cutaneous allodynia[92]. This highlights a need to further investigate the potential aetiology of the cutaneous allodynia observed with increased visceral adiposity. This link between visceral adiposity and cutaneous allodynia is curious as both cutaneous allodynia and obesity are risk factors for migraine chronification and disability[20, 47]. Given that increased visceral adiposity is associated with migraine, it could be expected that migraine patients have an altered hormonal profile, as is observed in abdominal obesity. In the context of IIH, recent evidence suggests that these patients have increased abdominal obesity relative to matched obese controls and that this abdominal fat plays a key role for the development of IIH[26]. It is yet unknown if adiposity itself is associated with headache in IIH.

Several studies have assessed the link between migraine and insulin resistance, a key metabolic feature of obesity. They demonstrated a degree of insulin resistance among fasted migraine patients as measured by the homeostatic assessment model (HOMA) compared to BMI and aged matched controls[93–95]. However, there is disagreement as to whether a CM or episodic migraine (EM) diagnosis is more highly associated with insulin resistance relative to controls. When directly comparing CM and EM, CM patients were demonstrated to be more insulin resistant than EM patients[93, 94]. These data could suggest similarities in the molecular underpinnings of CM and insulin resistance. In all these studies the migraine and control population were matched for BMI and none of the groups fell in the obese BMI category, and there were no changes in fasting glucose and glucose tolerance tests. The increased insulin resistance in CM could be linked to the aforementioned increased risk of CM in obesity. However, to our knowledge no studies have assessed insulin resistance in patients with concurrent obesity and migraine vs. patients with obesity. Such studies would help to delineate whether the increased insulin resistance in migraine is associated with concomitant obesity or a consistent part of the pathology. This increased insulin resistance in migraine is consistent with the observations of increased abdominal adiposity in migraine. In IIH, there is a severe insulin resistance phenotype, greater than that conferred by obesity[26]. Again, this increased insulin resistance is in keeping with increased abdominal obesity for a given BMI.

The adipokine leptin, which is raised in obesity, has also been associated with migraine. A single study has demonstrated that migraine patients have a higher serum leptin level, when adjusted for age, sex and BMI[88]. When split into migraine with aura (MA) and without aura (MO), MA patients had raised serum leptin whereas MO did not have raised leptin [88]. This likely links to the proposed increased risk of MA in women with obesity, particularly because women with obesity have higher serum leptin relative to males with obesity[89, 96]. Given the link between MA and CSD, the clinical findings of raised leptin in MA patients corroborate the pre-clinical finding that leptin experimentally alters CSDs and potentially provides a partial mechanism[87]. IIH patients have hyperleptinaemia in excess to that conferred by obesity[26]. However, in contrast to migraine where high leptin is associated with aura, there is currently little understanding about the frequency of aura in IIH patients. A single study reported 10 % of IIH patients have aura, much lower than the ~ 30 % in the general migraine population[97].

The studies presented demonstrate that there are similarities between obesity, migraine and IIH at the biochemical and anthropomorphic level (Table 2). However, whether the similarities are coincidence or contributors to headache parthogenisis requires further investigation. The present studies do not compare the endocrine profile of obesity related migraine to obesity. Such studies could help identity key differences that point to pathogenic moieties.

**Table 2:** Hormonal and anthropomorphic comparisons between obesity, migraine and IIH. Migraine with aura = MA, chronic migraine = CM, cutaneous allodynia = CA

Hormone	Migraine	Obesity	IIH
CGRP	Ictal ↑[1]	↑[2]	Not investigated
Amylin	Inter ictal ↑ [3]	↑[4]	Not investigated
Leptin	MA ↑ [5]	↑ [6]	↑↑ [7]
**Hormone state**			
Insulin resistance	CM↑ BMI Independent [8]	↑ [9]	↑↑ [7]
Central adiposity	↑ CA [10]	↑ body fat [9]	↑ Central obesity [7]

### The link between obesity and migraine, ***in-vivo*** behavioural and functional evidence

Several animal studies have explored the effects of obesity on migraine-like behaviour in mice, where photophobia and nocifensive behaviours have been assessed. It was demonstrated that both obese female and male mice develop a very modest basal photophobia that was accentuated by facial administration of capsaicin, a TRPV1 agonist and nociceptive[98, 99]. The degree of photophobia was weight dependent in high fat diet males, but not in female mice[98, 99]. This photophobic phenotype is replicated in genetically obese *ob*/*ob* mice on a normal diet, whereas lean mice on a high fat diet did not exhibit this behaviour, suggesting that excess adiposity confers a photophobic phenotype rather than a high fat diet[98]. However, the basal photophobia studies required large numbers of mice (n > 40) indicating a small effect that may not be functionally relevant.

There is also a suggestion of facial thermal cutaneous allodynia in obese mice, where obese female mice develop a stronger allodynia[100]. Paradoxically *ob/ob* mice show facial thermal cutaneous hypoalgesia, linking the presence of leptin to cephalic pain[101]. Although there is a suggestion of thermal cutaneous allodynia, there is currently no evidence of mechanical cutaneous allodynia in obese rodents. The capacity of obesity to modify mechanical cutaneous allodynia should be assessed given that mechanical cutaneous allodynia is readily treatable with migraine specific dugs in rodents[102]. The modest basal differences in behaviour that are modified by noxious stimuli suggest that excess adiposity increases the sensitivity of the rodent trigeminovascular system to noxious stimuli. Together, these data provide behavioural evidence that obesity potentiates migraine related nocifensive behaviour in rodents and thus provides pre-clinical evidence for the link between obesity and migraine.

### Functional alterations in the trigeminovascular system

Given the evidence that obesity adversely alters nocifensive behaviours associated with migraine and sensitivity in the trigeminovascular system, one would expect to observe obesity associated functional disturbances in these structures that could underlie these behavioural differences.

Cultured TG neurons derived from obese mice had a stronger calcium response to capsaicin than those derived from non-obese mice, indicating increased sensitivity to a noxious stimulus via TRPV1 agonism[98]. Although the mechanism underlying this was not delineated, it is feasible that it could be mediated by obesity associated inflammation. Pro-inflammatory cytokines promote membrane translocation of TRPV1 in nociceptive neurons, although this has yet to be demonstrated in the context of the TG in obesity[103]. Additionally, the capacity of obesity to alter CGRP release in TG has not been assessed.

It has been shown that dura mater from obese, insulin resistant rats released more CGRP basally and when stimulated with TRPA1 and TRPV1 agonists acrolein and capsaicin in the half cranial model [78, 104]. This corroborates the increased TRPV1 sensitivity of obese TG neurons to capsaicin, where *in situ*, TG neurons innervate the dura mater[98]. This increased response to noxious stimuli in the dura mater and TG may explain why capsaicin administration causes increased pain signalling, as indicated by increased activation in the external laminae of the trigeminal nucleus caudalis in obese mice, suggesting increased sensitivity to noxious stimuli [105].

Vasodilation in dural arteries is a proposed site for the origin of migraine pain[16]. Consequently, changes in sensitivity to vasodilators may alter the likeliness of developing a migraine. In obese rats, TRPA1 and TRPV1 agonists potentiate middle meningeal artery (MMA) blood flow, thus cause a vasodilation to a greater magnitude compared to control rats[78, 104]. Interestingly histamine and CGRP responses to MMA blood flow were comparable between obese and control rats, suggesting that the mechanism behind the difference is in part neuronal, rather than vascular. However, given the increase in basal dural CGRP release, dural arteries could become resistant to CGRPs vasodilatory functions. Together these studies suggest that the dural afferents of obese rats are more sensitive to noxious stimuli, thus promote a reflex associated with migraine.

**Fig. 1:**
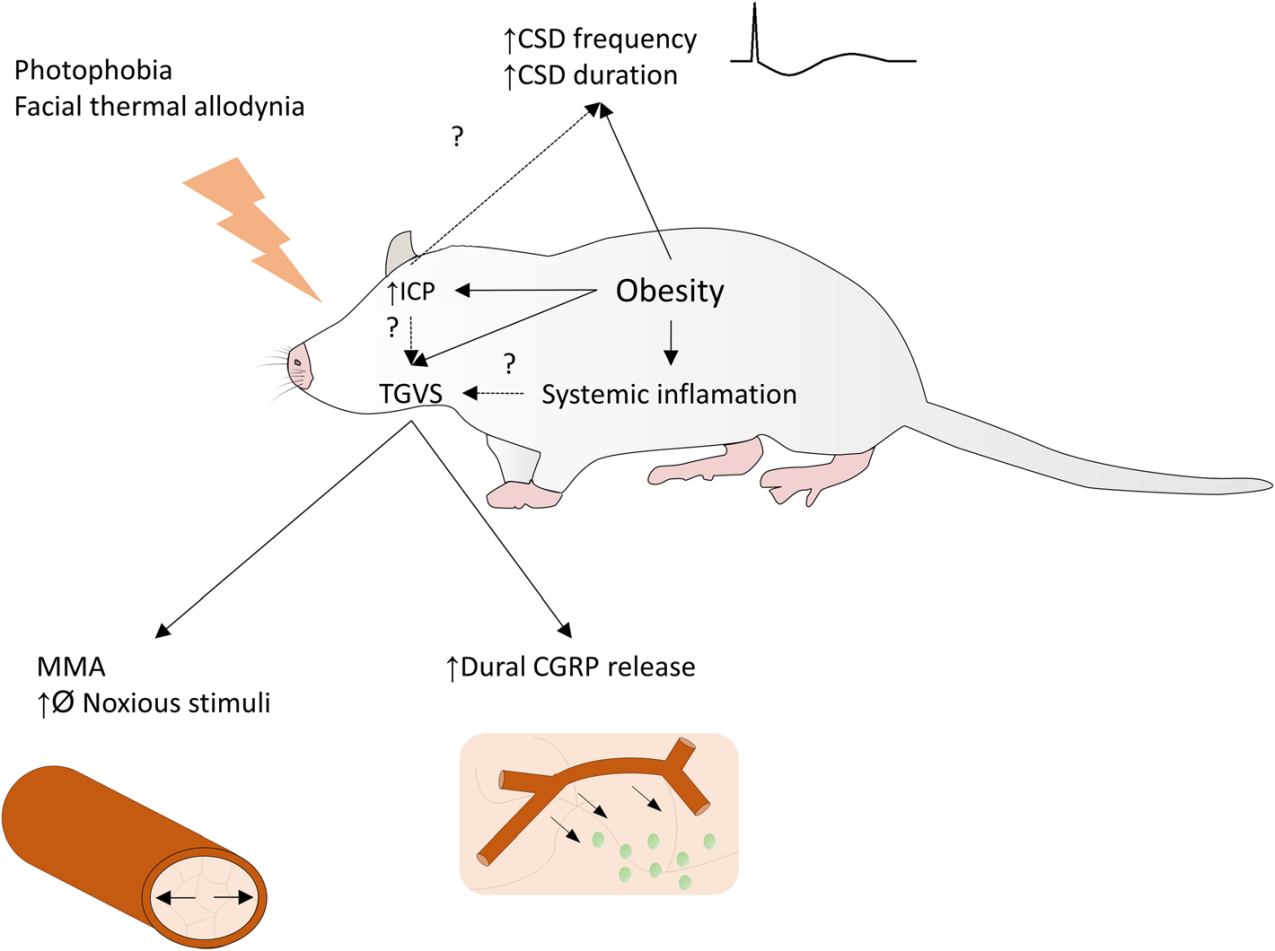
The effects of obesity on headache and the trigeminovascular system. Obesity has been demonstrated to affect several aspects associated with headache. Obesity alters headache-like behavior including increasing photophobia and thermal allodynia. This is associated with a series of physiological differences including increased cortical spreading depression (CSD) frequency. The trigemino-vascular system (TGVS) also is altered with obesity where the release of calcitonin gene related peptide (CGRP) from dura middle meningeal artery (MMA) calibre (Ø) display a greater response to noxious stimuli. The molecular underpinnings of these are unknown and it is unknown how obesity related systemic inflammation and intracranial pressure (ICP) alter the TGVS or headache in general

Together this body of evidence suggests that obesity increases the sensitivity of the trigeminovascular system to noxious stimuli, supporting the capsaicin induced behavioural abnormalities seen in obese mice. However, the present body of work does not pinpoint the molecular origin of these trigeminovascular disturbances in obesity and thus the increased noicfensive behaviour (Fig. 1). Additionally, the majority of work assessing the effects of obesity on migraine-like behaviour in rodents has been done in male rodents. Given that females make up the majority of migraine patients, assessing the effects of obesity on migraine-like behaviour in female rodents is essential.

### Obesity related factors and inflammation

Obesity is a disease of chronic systemic and tissue level inflammation and endocrine dysfunction; adipose tissue, the liver and pancreatic islets demonstrate obesity specific inflammation[13–15]. This inflammation is caused in part by the secretion of cytokines from tissue resident and infiltrating macrophages[13–15]. Given the systemic and local inflammation, and endocrine dysfunction in obesity, it is plausible that obesity is affecting the physiology of the trigeminovascular system. This is particularly important as the TG and dura mater are outside of the blood brain barrier (BBB) and thus exposed to the circulating inflammatory milieu associated with obesity [70]. Although the effects of obesity on inflammation and the endocrinology of the trigeminovascular system remain unclear, investigations into the effect of induced inflammation can provide insight into this under researched area and highlight future research avenues.

IL-1β is a classic pro-inflammatory cytokine and potent hyperalgesic that is raised at the serum and tissue level in both obese humans and rodents[106]. Functionally, IL-1β has the capacity to increase the membrane potential of small TG neurons. These nociceptive neurons demonstrated more spiking behaviour upon IL-1β administration through altered K^+^ conductance[107, 108]. This suggests that the nociceptive neurons become more sensitive to stimuli, thus nociception. This study also demonstrated that peripheral inflammation induced upregulation of IL-1β on TG satellite glial cells and upregulation of IL-1β receptor on TG neurons[107]. This suggests that paracrine signalling between activated satellite glia and TG neurons could drive increased sensitivity of the TG nociceptors. The altered K^+^ conductance in inflammation is likely directly linked to migraine pathology. The K^ATP^ channel opener levkromakalim has been demonstrated to induce migraine in humans and migraine-like behaviour in mice, where K^ATP^ channel opening is hypothesised to be a common pathway in migraine pain[102, 109]. Given this evidence, it is feasible that both the dura mater and TG are exposed IL-1β in the context of obesity. However, in the context obesity, it is unknown whether there are tissue level increases of IL-1β in migraine related structures.

TNF-α is another pro-inflammatory cytokine associated with obesity that induces hyperalgesia[110]. However, rather than being raised systemically, TNF-α expression in obesity is increased at the tissue level in tissues such as adipose tissue and the liver among others[111, 112]. As such, in the context of pro-inflammatory stimuli in obesity, it is conceivable that TNF-α expression is raised in the trigeminovascular system. TNF-α causes CGRP release in cultured TG neurons in a dose dependent manner[113]. Furthermore, TNF-α derived from macrophages has been demonstrated to increase cephalic allodynia in a rat TG trauma model, which is treatable via TNF-α sequestration with etanercept[114]. This demonstrates that activated macrophages have the capacity to promote nociception in the TG via TNF-α mediated paracrine signalling. Given that TNF-α expression is increased at the tissue level in obesity, particularly in tissue resident macrophages, it could be hypothesised that obesity causes an upregulation of TNF-α in TG macrophages and promote nociception[111, 112]. Further work is required to link inflammation and the TG in the setting of obesity.

Together, these data highlight that nociceptive cytokines known to be raised in obesity have the capacity to promote nociception at the TG. However, the expression of pro-inflammatory cytokines has yet to be assessed in an obese migraine model and thus should be a focus of future research.

### Intracranial pressure, obesity and headache

In obesity, there is a growing body of evidence that increased BMI is associated with increased ICP [115, 116]. Indeed, body fat percentage and ICP positively correlate in a mixed neurological patient cohort[117]. Furthermore, in patients with IIH it has been demonstrated that truncal adiposity positively correlates with ICP[118]. In addition, obese Zucker rats have raised ICP[119]. Together these data link adiposity to raised ICP, although the potential mechanisms underlying this are unelucidated. However, it is controversial as to whether ICP directly contributes to headache.

In IIH, weight loss and concurrent ICP reduction improves headache. Consequently, it could be suggested that deranged ICP dynamics have the potential to damage components of the trigeminovascular system causing headache or central sensitisation to pain[40]. Indeed, the structures associated with migraine are within the central nervous system, where they are under the mechanical influence of ICP and any changes in ICP could cause pain. Consistent with this, cortical tissue from IIH patients demonstrated pathological features such as astrogliosis, BBB leakage and abnormal mitochondrial morphology, which correlate with pathological ICP waveforms [120–122]. Furthermore, ICP may alter central sensitivity to pain: it has been demonstrated that ICP correlates with cutaneous allodynia and that reducing ICP causes a reduction in cutaneous allodynia in IIH patients[123, 124]. This suggests that raised ICP could lower the threshold for a migraine attack. Direct evidence of ICP influencing and potentially damaging the trigeminovascular system is lacking. Further evidence that raised ICP in the context of obesity could contribute to migraine is that one study has demonstrated that 10 % of chronic migraine patients met the diagnostic criteria for IIH without papilledema (IIHWOP), where IIHWOP is otherwise considered a rare presentation of IIH[125].

Given the evidence that headache in IIH can be treated with erenumab, this suggests that either the pressure related headache in IIH is migraine, or that the IIH headache and migraine have differing pathophysiology and present in the same phenotype. Perhaps in agreement with this, a resent in vivo paper demonstrated that different rodent migraine triggers can signal through differing pathways[126].

Obesity mediated ICP increases could also alter CSD frequency: it is established that pathologically raised ICP is associated with increased CSD frequency in experimental and clinical settings[127, 128]. Indeed, obese Zucker rats have been demonstrated to have both raised ICP and increased CSDs, although it has yet to be specifically demonstrated that obesity mediated ICP increases alter CSD[87, 129]. Thus, obesity mediated ICP increases could explain the increased MA in obese females, although the apparent reduction in aura in IIH confuses the situation. Further research is required to understand the link between raised ICP and aura.

With all of this however, it is unclear as to what degree an ICP increase may increase migraine sensitivity or directly cause a headache. This should thus be an avenue for future research. Additionally, further investigation into the ICP phenotype in both lean and obese migraine patients is important to delineate the role ICP has in migraine-like headache.

## Conclusions

Migraine and obesity affect large numbers of individuals around the globe. A clear link between these diseases exists, both through epidemiology and evidenced though clinical intervention. It is apparent that obese migraine patients exhibit a more disabling phenotype than non-obese migraineurs. Furthermore, raised ICP, as present in IIH, is associated with obesity and headache. The pre-clinical evidence demonstrates that obese rodents show migraine-like behaviour and have functional abnormalities in the migraine related structures. However, these pre-clinical studies fail to identify the molecular underpinnings linking obesity to migraine or IIH, understanding of this could stimulate the development of therapeutics for obesity related migraine.
